# Titanium dioxide nanoparticle-modified screen-printed electrode for erlotinib determination

**DOI:** 10.5599/admet.3437

**Published:** 2026-08-09

**Authors:** Pooja Das Manjulabhai, Sruthi Mundangadan, Maria Paul, Srinivedha Lal, Namitha Ramalal, Mariya Anto, Dhanya Gangadharan

**Affiliations:** Department of Biotechnology, Sahrdaya College of Engineering and Technology, Affiliated to APJ Abdul Kalam Technological University, Kodakara, Thrissur, Kerala, India

**Keywords:** Electrochemical sensing, differential pulse voltammetry, therapeutic drug monitoring

## Abstract

**Background and purpose:**

Erlotinib (ERL), a first-generation epidermal growth factor receptor tyrosine kinase inhibitor drug that is used in non-small cell lung cancer treatment, requires precise therapeutic drug monitoring owing to its narrow therapeutic window and significant inter-patient pharmacokinetic variability; existing chromatographic methods, while robust, are resource-intensive and incompatible with point-of-care settings, necessitating the development of simpler, cost-effective electroanalytical alternatives.

**Experimental approach:**

A titanium dioxide nanoparticle-modified screen-printed electrode (TiO_2_NP@SPE) was fabricated via a facile single-step modification and comprehensively characterized by scanning electron microscopy - energy dispersive X-ray Analysis, Fourier transform infrared spectroscopy, X-ray diffraction and electrochemical impedance spectroscopy; electrochemical performance was evaluated by cyclic voltammetry and differential pulse voltammetry, and analytical validation was performed in human serum using the standard addition method.

**Key results:**

The TiO_2_NP@SPE demonstrated markedly enhanced electron transfer kinetics over the bare SPE, yielding a well-defined linear response with a competitive limit of detection, high sensitivity, and intra- and inter-day serum recoveries within acceptable precision limits, alongside excellent selectivity against physiologically relevant interferents and structurally related anticancer agents.

**Conclusion:**

This work developed a disposable screen-printed electrode sufficient to achieve sensitive, selective, and accurate quantification of ERL in human serum using TiO_2_NP@SPE as a promising, clinically translatable platform for point-of-care therapeutic drug monitoring in oncology; future efforts should address the elevated detection limit relative to the bare electrode and extend validation to real patient plasma samples to fully confirm clinical applicability.

## Introduction

Lung cancer, particularly non-small cell lung cancer (NSCLC), remains one of the most prevalent and lethal malignancies worldwide. Erlotinib (ERL), an epidermal growth factor receptor (EGFR) tyrosine kinase inhibitor, was approved by the U.S. Food and Drug Administration (FDA) in November 2004 for the treatment of metastatic NSCLC. At the standard therapeutic dose of 150 mg *per* day, ERL steady-state trough plasma concentrations in NSCLC patients exhibit marked inter-individual variability, typically ranging from approximately 0.73 μM (315.6 ng mL^-1^) to 10.43 μM (4480 ng mL^-1^), with a minimum effective threshold of around 1.16 μM (500 ng mL^-1^) proposed for adequate EGFR inhibition [1-3]. This wide pharmacokinetic variability, coupled with the narrow margin between therapeutic and toxic concentrations, underscores the clinical necessity for routine therapeutic drug monitoring (TDM) and an analytical platform capable of quantifying ERL across this entire concentration span [4]. Recent clinical models continue to confirm that severe inter-individual exposure and adverse outcomes, such as interstitial lung disease, are strongly driven by these variable plasma concentrations, necessitating robust patient-centric monitoring [5-7]. Despite its therapeutic efficacy, erlotinib is associated with significant adverse effects including folliculitis, diarrhoea, dry skin, and fatigue, which may necessitate dose reductions or early discontinuation [8]. Consequently, therapeutic drug monitoring (TDM) is critical for optimizing dosing, improving therapeutic outcomes, and minimizing toxicity [9-12].

Conventional methods for ERL quantification, including high-performance liquid chromatography (HPLC), liquid chromatography-mass spectrometry (LC-MS), liquid chromatography-tandem mass spectrometry (LC-MS/MS) and others are characterized by high sensitivity but require sophisticated instrumentation, trained personnel, lengthy sample preparation and hazardous solvents [13-18]. Immunoassay-based platforms, while offering some multiplexing advantages, suffer from cross-reactivity, interference from endogenous biomolecules, and lower specificity [19-21]. These limitations underscore the need for rapid, low-cost, and field-deployable analytical platforms. Electrochemical methods have emerged as powerful alternatives owing to their simplicity, cost-effectiveness, miniaturisability and inherent compatibility with point-of-care settings [22-25]. Indeed, state-of-the-art voltammetric sensor development has aggressively targeted tyrosine kinase inhibitors using composite matrices to meet clinical accuracy demands in complex biological fluids [26-29]. Among these, disposable SPEs are particularly attractive because of their affordability, portability, and suitability for mass production [29-32].

Titanium dioxide nanoparticles (TiO₂NP) are widely employed in electrochemical sensing due to their large surface area, excellent biocompatibility, chemical stability, and pronounced electrocatalytic properties [33-34]. Modern nano-interfaced designs increasingly leverage the high surface-to-volume ratio and catalytic properties of TiO₂ on SPEs to expedite heterogeneous electron transfer rates and boost trace-level sensor sensitivity [35]. TiO₂NPs have been shown to enhance electron-transfer kinetics and improve analyte adsorption at electrode surfaces, leading to improved detection performance. Among the metal oxide nanomaterials explored for electrochemical sensing, including ZnO, CeO₂ and Fe₃O₄, TiO₂ was specifically selected in this work for three key reasons. First, anatase-phase TiO₂ exhibits exceptional chemical stability across the neutral pH range employed in physiological sensing (pH 7.4), remaining insoluble and structurally intact in phosphate buffer, unlike ZnO which undergoes surface dissolution under similar conditions [36]. Second, TiO₂ exhibits well-documented electrocatalytic activity toward the oxidation of aromatic organic compounds, attributable to its abundant surface hydroxyl groups and high adsorptivity for π-electron-rich molecules such as erlotinib [37,38]. Third, TiO₂ is established as non-toxic and biocompatible, a critical requirement for sensors intended for clinical serum analysis, where cytotoxic modifier residues could compromise sample integrity and patient safety.

In the present study, we report the fabrication and characterization of a TiO₂NP-modified SPE (TiO_2_NP@SPE) and evaluate its application for the ultrasensitive electrochemical determination of erlotinib. The modified sensor achieved substantially higher sensitivity and an improved electroactive surface area compared to the unmodified electrode, while shifting the linear range to a range more relevant to clinical TDM concentrations, with excellent selectivity and reproducibility in human serum samples, demonstrating its suitability for TDM in clinical practice.

## Experimental

### Reagents and materials

ERL was received as a gift from a pharmaceutical company (Hyderabad, India). TiO_2_ nanoparticles (anatase, ≥99 %, particle size < 30 nm) were procured from (CAS No: 13463-67-7, NCZD2902, Nanochemazone, Canada). A 1 mM ERL stock solution was prepared in methanol and stored at 4 °C in a dark container. All reagents were of analytical reagent grade. Phosphate buffer (PB, 0.1 M, pH 7.4) was prepared using KH_2_PO_4_ and Na_2_HPO_4_ in deionized (DI) water and used as the supporting electrolyte throughout. Screen-printed electrodes were fabricated using conductive carbon ink (Code No: 050-Sun Chemical C2030519P4, Bangalore), conductive silver ink (Code No: 060; Siltech Corporation Inc., Bangalore), and conductive Ag/AgCl paste (CAS No: 7783-90-06, NCZ-SPI-104, Nanochemazone, Canada) on 25 μm polyethylene terephthalate (PET) sheets.

### Instrumentation

Electrochemical experiments were performed using a DY2300 Bipotentiostat (Digi Ivy, USA). SEM-EDAX analysis was performed using a Gemini SEM 300 (Carl Zeiss, Germany). XRD was recorded using an Aeris Research benchtop X-ray diffractometer (Malvern PANalytical, UK). SPEs were directly analysed by FTIR spectroscopy using Shimadzu IRSprit-X (Serial no. A230962). EIS was performed using the CHI6005E Electrochemical Workstation of Central Instrumentation Facility of IIT Palakkad.

### Fabrication of the TiO₂ nanoparticles-modified screen-printed electrode

Bare SPEs were fabricated by screen-printing a silver conductive layer followed by a carbon layer and an Ag/AgCl reference electrode layer on PET sheets, as described in our previous study [39]. For surface modification, TiO₂NPs were thoroughly mixed with carbon paste at a weight ratio of 1:4 (TiO₂NPs : carbon paste) to obtain a homogeneous composite. The resulting paste was then screen-printed over the working electrode surface and subsequently dried at 60 °C to ensure complete solvent evaporation and firm adhesion of the composite layer. The modified electrode (TiO₂NP @SPE) was then conditioned by running 10 consecutive cyclic voltammograms in 0.1 M PB (pH 7.4) prior to use. Conditioning was performed to stabilize the electrode surface and remove loosely adsorbed nanoparticles, ensuring reproducible baseline responses.

### Electrochemical characterization

The electrochemically active area of the TiO_2_NP@SPE was determined using cyclic voltammetry in 5 mM [Fe(CN)_6_]^3-/4-^ with 0.1 M KCl at scan rates ranging from 2 to 200 mV s^-1^, applying the Randles-Ševčík equation. Scan rate studies for ERL oxidation were conducted with 15 μM ERL in 0.1 M PB (pH 7.4) at scan rates from 5 to 200 mV s^-1^ (potential window: 0.2-0.9 *V*). The nature of the electrode process (adsorption *vs.* diffusion-controlled) was determined from plots of *I*_pa_ *vs. v* and *I*_pa_ *vs. v*^-1/2^. The supporting electrolyte pH was maintained at 7.4 throughout, as this value corresponds to the physiological pH of human serum and was established as the optimal condition for ERL oxidation at SPE in our previous study [39]; since TiO₂NP modification does not alter the fundamental redox mechanism of ERL as confirmed by the identical irreversible oxidation behaviour and consistent peak potential observed in the scan rate study and electrode kinetics below so a separate pH optimization on the modified electrode was not performed

### Sensitivity studies and calibration

DPV measurements were performed in 0.1 M PB (pH 7.4, as described in the Results and discussion, section Sensitivity studies: calibration and detection limits) over a potential window of 0 to 0.8 V, with ERL concentrations ranging from 15 to 65 μM. DPV parameters were: pulse amplitude 50 mV, potential increment 50 mV, pulse width 50 ms, and pulse period 100 ms, which were optimized in previous experiments [39]; the parameters that afforded the maximum peak current and best peak resolution were selected. The limit of detection (LOD) and limit of quantification (LOQ) were calculated as 3*σ*/*m* and 10*σ*/*m*, respectively, where *σ* is the standard deviation of the current response of 10 replicate measurements of the blank (0.1 M PB, pH 7.4, in the absence of ERL) and *m* is the slope of the calibration curve.

### Selectivity studies

The selectivity of the TiO_2_NP@SPE was evaluated in the presence of common biological interferents includeing inorganic salts (Mg^2^⁺, Na^2^⁺, K⁺, Ca^2^⁺), sugars (glucose, sucrose, maltose), dopamine, urea, uric acid, fluoroquinolones (moxifloxacin (MOX), levofloxacin (LEV)), antifungal drug tinidazole (TNZ), acetaminophen (ACT), pantoprazole (PNP), metformin (MET) and structurally similar anticancer drugs (imatinib (IMT), capecitabine (CAP), sunitinib (STB), dasatinib (DSB)). Voltammetric responses were recorded in the presence of each interferent at concentrations that mimic physiological conditions, together with 22 μM ERL. Interferent concentrations were selected to reflect physiological levels in human serum or to represent a 1000-fold excess over the ERL concentration tested, whichever was higher.

### Reproducibility, stability and recovery studies

Repeatability was assessed by comparing the DPV responses of 8 runs on a single modified electrode with 55 μM ERL. Reproducibility was assessed by comparing the DPV response of 6 individually prepared TiO_2_NP@SPE electrodes to 55 μM ERL. Stability was evaluated over 30 days on electrodes stored at room temperature. Recovery studies were conducted on two human serum samples (obtained from healthy volunteers) spiked with ERL at three concentrations (33, 44 and 55 μM) using the standard addition method. Both intra-day (*n* = 6) and inter-day analyses were performed. Student's *t*-test (95 % confidence, degrees of freedom *(df* = 5)) was applied to assess statistical significance.

### Statistical data analysis

Experimental data were collected in triplicate and analysed using the Microsoft Excel Data Analysis ToolPak. One-way analysis of variance (ANOVA) in Origin 2026b [40] was used to determine whether the ERL concentrations identified using the developed approach differed significantly.

## Results and discussion

### Fabrication of TiO₂ nanoparticles modified screen-printed electrode

**Figure 1.**
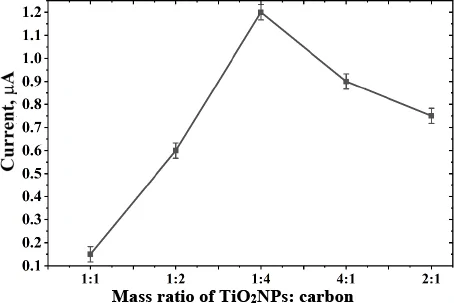
Effect of TiO₂NP concentration on sensor response. The graph illustrates the relationship between the differential peak current (Δ*I*_p_) and the TiO₂NP: Carbon mass ratio in the presence of 15 μM ERL. Error bars represent the standard deviation for n=3 measurements

To fabricate the nanocomposite, TiO₂NP: carbon paste was tested in various ratios (1:1, 1:2, 1:4,4:1,2:1). A ratio of 1:4 was found to give the best results for the determination of 15 μM ERL (Figure 1). At ratios higher than 1:4 (*e.g.*, 1:2 and 1:1), the higher TiO₂ content increased electrode resistance due to the semiconducting nature of TiO₂, decreasing conductivity; at lower ratios (2:1 and 4:1), insufficient TiO₂ reduced the available electroactive surface area. The enhanced current observed with this ratio can be attributed to the increased electroactive surface area and improved interfacial electron transfer facilitated by the TiO₂ nanoparticles, which provide an efficient electron-transfer network, while their large surface area increases the number of electroactive sites. This ratio ensures optimal dispersion of TiO₂NPs, preventing aggregation and maintaining accessibility to catalytic sites, supporting the structural integrity of the nanocomposite [41,42].

### Morphological and structural characterization of TiO₂ nanoparticles modified screen-printed electrode

**Figure 2.**
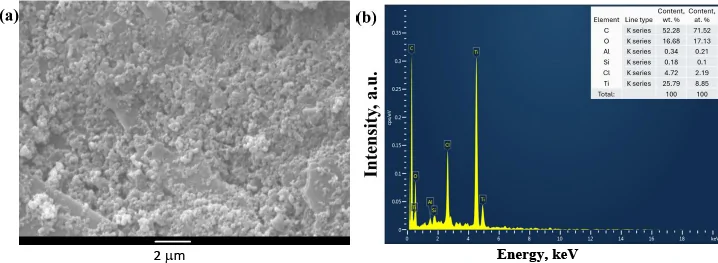
(a) SEM images and (b) EDAX spectrum with elemental composition (inset) confirming successful TiO₂NP deposition on the electrode surface

The surface morphology of the TiO_2_NP@SPE was examined by SEM (Figure 2a). The micrograph reveals a rough, granular surface with well-dispersed TiO_2_ nanoparticles. This morphology is markedly different from the relatively smooth topography typical of a bare SPE, suggesting a successful increase in the electroactive surface area. EDAX analysis (Figure 2b) confirmed the successful deposition of TiO₂NP, with titanium appearing as a prominent constituent (25.79 wt.%) [43,44]. The spectrum also shows significant peaks for carbon (52.28 wt.%) and oxygen (16.68 wt.%), corresponding to the graphite ink and the oxide phase of the nanoparticles, respectively. Trace amounts of aluminium, silicon and chlorine were detected, which are consistent with the composition of the PET substrate and the conductive ink binder.

**Figure 3.**
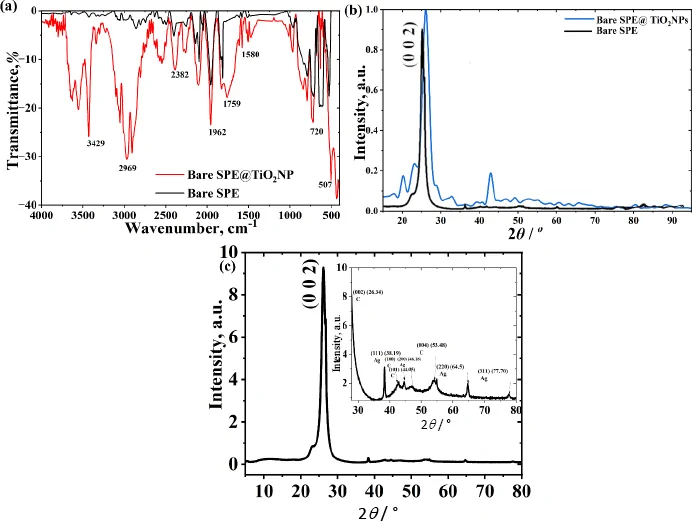
Structural and chemical characterization of the modified electrodes. (a) FTIR spectra of the bare screen-printed electrode (Bare SPE, black) and the electrode modified with titanium dioxide nanoparticles (TiO_2_NP@SPE, red), showing characteristic vibrational bands for the functional groups and the Ti-O-Ti stretching at 507 cm^-1^. (b) XRD patterns comparing the crystalline structure of the TiO₂NP (red) and the modified SPE (blue), where the modified SPE shows a dominant peak at 26° associated with the carbonaceous support. (c) Detailed XRD spectrum of the modified material highlighting the sharp (002) diffraction peak of graphitic carbon at approximately 26°, with an inset displaying the broader diffraction range and higher-order peaks (100, 101, 102, 110, *etc*.) indexed to the electrode's substrates like silver and carbon

The FTIR spectra of the bare SPE and TiO_2_NP@SPE (Figure 3a) were acquired to identify the functional groups, and vibrational modes present in each material and to confirm successful nanoparticle incorporation. The bare SPE (black line) displays characteristic peaks at 1580 cm^-1^, corresponding to C=C aromatic stretching vibrations of the graphitic carbon matrix, confirming the carbonaceous nature of the electrode substrate [41]. Upon modification with TiO₂NP (red line), a prominent new absorption band appears at 507 cm^-1^, which is entirely absent in the bare SPE spectrum and is unambiguously assigned to the Ti-O and Ti-O-Ti stretching vibrations characteristic of the anatase phase of TiO₂ [43,44]. The exclusive appearance of this band in the modified electrode spectrum provides definitive spectroscopic confirmation of successful TiO₂NP deposition onto the electrode surface without phase transformation. Additionally, the TiO_2_NP@SPE displays a broad band at 3429 cm^-1^ attributable to O-H stretching vibrations of surface hydroxyl groups and adsorbed water molecules on the TiO₂ surface [44,45], which are electrochemically significant as they facilitate analyte adsorption. The band at 2969 cm^-1^ is consistent with asymmetric C-H stretching vibrations of aliphatic hydrocarbon groups originating from the organic polymeric binder present in the conductive carbon ink matrix [46]. The absorption at 2382 cm^-1^ is attributable to atmospheric CO₂, a well-known artifact commonly observed in attenuated total reflectance -Fourier transform infrared (ATR-FTIR) measurements [46]. The peak at 1759 cm^-1^ is assigned to C=O stretching of ester or carbonyl groups within the polymeric binder resin of the conductive ink [47]. Collectively, the FTIR data confirm the chemical identities of both the carbon substrate and the TiO₂ modifier and unambiguously establish that the nanoparticle modification introduces new Ti-O bonding while preserving the electrode's graphitic carbon structure.

The structural phase, crystallinity, and crystallite size of the synthesized materials were characterized by XRD (Figure 3b). The TiO₂ NPs display sharp, well-defined diffraction peaks at 2θ ≈ 25.3, 37.8, 48.0, 53.9, 55.1 and 62.7, corresponding to the (101), (004), (200), (105), (211) and (204) crystallographic planes of the anatase phase (JCPDS No. 21-1272), confirming phase-pure anatase with no detectable rutile or brookite impurities. The sharpness and high intensity of these reflections indicate good crystallinity of the nanoparticles. The average crystallite size of the TiO₂ NPs was determined using the Debye-Scherrer equation (1):



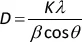

(1)


where *K* = 0.9 (dimensionless shape factor), *λ* = 0.15406 nm (Cu Kα radiation), β is the full width at half maximum (FWHM) of the diffraction peak in radians, and *θ* is the Bragg diffraction angle. Applying this to the most intense anatase reflection, the (101) peak at 2*θ* = 25.3° (FWHM = 0.55°) yields an average crystallite size of *D* = 14.8 nm. This value is in close agreement with the manufacturer-stated particle size of <30 nm, with the difference being consistent with the well-established distinction between XRD-derived coherent scattering domain size and physical particle dimensions measured by electron microscopy.

The TiO₂ anatase peaks remain clearly visible in the modified SPE spectrum alongside the graphitic carbon signal, and the crystallite size derived from the (101) reflection in the modified SPE (*D* = 13.6 nm, FWHM ≈ 0.60°) is marginally smaller than that of the bare TiO₂ NPs, which may be attributed to slight lattice strain or confinement effects upon immobilization of the nanoparticles onto the electrode surface. Collectively, the retention of all characteristic anatase reflections, along with the graphitic carbon peak, confirms the successful integration of TiO₂ NPs onto the electrode surface without inducing any phase transformation of the nanoparticles [46,47]. In the bare SPE, a dominant high-intensity peak at 2*θ* = 26.5° is observed, corresponding to the (002) plane of graphitic carbon from the SPE substrate [48,49]. Applying the Debye-Scherrer Equation (1) to this (002) reflection (FWHM = 0.20°) yields a graphitic stacking crystallite height of *L*_c_ ≈ 40.8 nm, indicative of well-ordered, highly crystalline graphite layers within the SPE [50,51].

### Electrochemical active area determination

**Figure 4.**
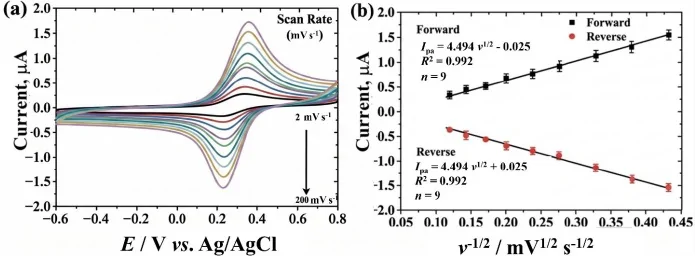
Electrochemical characterization of the modified electrode. (a) Cyclic voltammograms of TiO_2_NP@SPE recorded in 5 mM Fe[(CN)_6_]^3-/4-^containing 0.1 M KCl at various scan rates ranging from 2 to 200 mV s^-1^. (b) Linear regression plots of the anodic (*I*_pa_) and cathodic (*I*_pc_) peak currents versus the square root of the scan rate ʋ^1/2^, demonstrating a diffusion-controlled redox process

The electrochemically active area (ECA) of the TiO_2_NP@SPE was calculated using the Randles-Ševčík equation applied to [Fe(CN)_6_]^3-/4-^ cyclic voltammograms at varying scan rates (Figure 4). The anodic and cathodic peak currents varied linearly with the square root of scan rate (*R^2^* = 0.99), confirming diffusion-controlled mass transfer. Based on the slope extracted from the linear regression, *I*_pa_ = 4.494 *v*^1/2^ and assuming standard parameters for a 5 mM ferricyanide solution (*n* = 1, *D* = 7.6×10^-6^ cm^2^ s^-1^), the calculated electrochemically active area of the TiO_2_NP@SPE was found to be 0.0383 cm^2^. All further electrochemical experiments were conducted at a scan rate of 50 mV s^-1^.

The calculated ECA of 0.0383 cm^2^ for the TiO_2_NP@SPE is primarily attributed to the synergistic effect of the nanomaterial modification on the carbon substrate. While the geometric area of fabricated screen-printed electrodes is typically small (approx. 0.0314 cm^2^ for a 2 mm diameter), the integration of TiO_2_ nanoparticles enhances the ECA by providing a higher density of electronic states and increasing the surface roughness factor [52,53].

### Scan rate study and electrode kinetics

**Figure 5.**
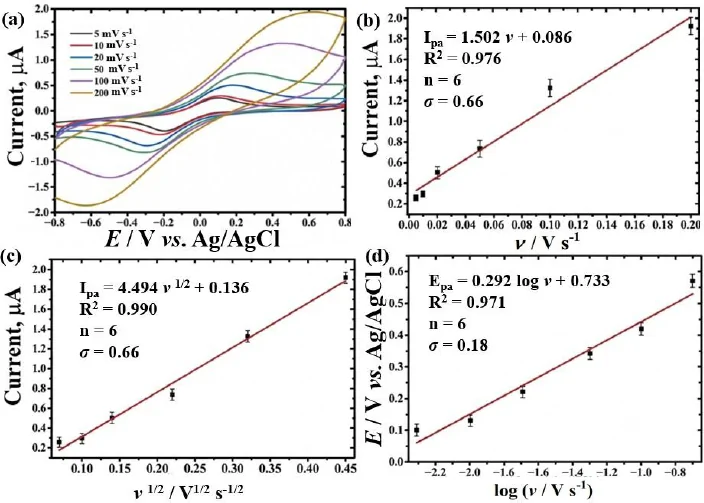
Electrochemical characterization of the modified electrode: (a) Cyclic voltammograms at varying scan rates; (b) Linear plot of anodic peak current (*I*_pa_) *vs. v* (*R*^2^ = 0.976); (c) Linear plot of *I*_pa_ *vs. v*^1/2^ (*R*^2^ = 0.990) indicating a diffusion-controlled process; and (d) Linear relationship between peak potential (*E*_pa_) and log *v* used to calculate the kinetic parameters (*αn*) *via* Laviron’s Equation (2)

The electrochemical oxidation mechanism of ERL on TiO_2_NP@SPE was investigated by CV at scan rates from 5 to 200 mV s^-1^ in the presence of 15 μM ERL in 0.1 M PB (pH 7.4) (Figure 5a). As the scan rate increased, the anodic peak potential shifted positively, indicating an irreversible oxidation process. The linear relationship between *I*_pa_ and *v*^1/2^ (Figure 5c, *R*^2^ = 0.99) established that the electrode process is diffusion-controlled, consistent with the behaviour previously observed on the bare SPE.

The scan rate was increased, and the linear relationship between the peak potential and the logarithm of the scan rate (Figure 5d) can be described by Equation (2). *E*_pa_ = 0.292 log *v* + 0.086, (*R*^2^ = 0.971, *σ* = 0.18, *n* = 6). *E*_pa_ for an irreversible process can be defined by the Laviron Equation (2) [54,55]:



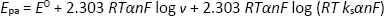

(2)


where *k*_s_ is the charge transfer rate constant. Taking *T* = 298 K, *R* = 8.314 J K^-1^ mol^-1^ and *F* = 96480 C mol^-1^, the value of *αn* can be calculated to be 0.20 from the slope of *E*_pa_ *vs.* log *v* (0.292). Where α is the transfer coefficient and *n* is the number of electrons transferred during the electrode process. Using the Bard-Faulkner equation (3) [56] for the irreversible process, the charge transfer coefficient (*α*) was calculated to be 0.40.



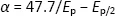

(3)


By substituting the value of *E*_p_ and *E*_p/2_, the potential where the current is at half the peak value. The number of electrons *n* was found to be ~1 for oxidation, which agrees with the previously reported results for ERL. Further, from the intercept of the *E*_pa_ versus v curve by extending to the vertical axis at *v* = 0, the value of *E*⁰ (redox potential) was found to be 0.733 V. *k*_s_ is the apparent charge transfer rate constant of the reaction and was calculated to be approximately 1.2×10⁻^2^ s^-1^ [57].

The incorporation of TiO_2_NP enhanced the electron transfer kinetics at the electrode surface relative to the bare SPE, as evidenced by improved peak definition and higher peak currents. Electrode modification enhances current sensitivity and lowers overpotential, but it does not change the intrinsic redox stoichiometry of the analyte, which is governed by the molecule's chemical structure. According to Laviron's theory, the calculated value of *n* reflects only the electrons involved in the rate-determining step, meaning subsequent rapid transfers will not increase the observed kinetic electron count. Consequently, the modification improves the efficiency of the interface without altering the fundamental number of electrons the molecule is thermodynamically capable of transferring [58,59].

### Probable electrochemical oxidation mechanism

**Figure 6.**
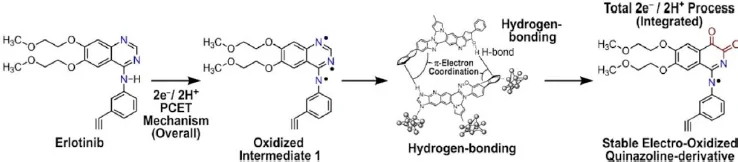
Schematic representation of the electrocatalytic oxidation mechanism of erlotinib on a TiO_2_ modified electrode surface through a sequential 2 e^-^/ 2 H^+^ PCET mechanism

To further elucidate the pathway underlying the electrocatalytic performance of the developed sensor, a molecular oxidation mechanism for ERL is proposed based on our experimental kinetics (as shown in Figure 6) and the established quinazoline electrochemistry literature by Bakirhan *et al.* [60]. Structurally, ERL consists of an electron-rich quinazoline core substituted with an aniline moiety and ethoxy side chains [60]. The electrochemical oxidation occurs predominantly at the nitrogen heteroatoms of the central azaheterocyclic quinazoline ring system. The overall process is governed by a proton-coupled electron-transfer mechanism involving two electrons and two protons (2e-/2H+), proceeding in two distinct sequential steps. ERL undergoes an initial slow, irreversible loss of one electron (e^-^) and one proton (H^+^) at the nitrogen site to generate a highly reactive radical intermediate. This is fully supported by our application of the Laviron and Bard-Faulkner Equations (2) and (3), which yielded an electron count of *n* approximately 1 for the reaction. The unstable radical intermediate rapidly loses a second electron (2^nd^ e^-^) and a second proton (2^nd^ H^+^) to form a stable electro-oxidized quinazoline-derivative product.

The integrated TiO_2_ nanoparticles play a dual electrocatalytic role in this pathway. First, the abundant surface hydroxyl -OH groups identified in our FTIR spectra facilitate strong hydrogen-bonding interactions with the nitrogen atoms of the incoming ERL. Second, the high density of states on the nanostructured anatase surface coordinates seamlessly with the π -electron-rich aromatic rings of the drug. This localized surface concentration pre-orients the molecules, substantially lowering the interfacial charge-transfer resistance *R*_ct_ decreased from 250 to 110 Ω and leading to the enhanced current responses observed [61,62].

### Electrochemical characterization of the modified electrode

**Figure 7.**
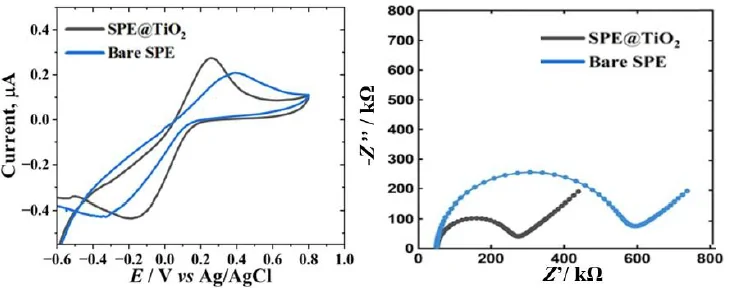
Electrochemical characterization of bare SPE and TiO_2_NP@SPE. (a) Cyclic voltammograms and (b) Nyquist plots of the bare SPE (blue line) and TiO_2_NPs-modified SPE (black line) recorded in 5 mM Fe[(CN)_6_]^3-/4-^ containing 0.1 M KCl. CV scan rate: 50 mV s^-1^. EIS measurements were performed over a frequency range from 0.1 Hz to 100 kHz at an amplitude of 5 mV

The electrochemical behaviour of the bare SPE and the TiO_2_NPs-modified SPE was investigated using CV and EIS. The study was conducted in a probe solution containing 5 mM [Fe(CN)_6_]^3-/4-^ and 0.1 M KCl. As illustrated in Figure 7a, both electrodes exhibited characteristic reversible redox peaks associated with the [Fe(CN)_6_]^3-/4-^ couple. Significant differences were observed following surface modification. The bare SPE (blue line) showed a broader peak potential separation (Δ*E*_p_) and a lower anodic peak current, *I*_pa_ = 0.21 μA. Upon modification with TiO_2_NP (black line), the anodic peak current increased to approx. 0.28 μA. Furthermore, the Δ*E*_p_ decreased, with the anodic peak shifting toward a more negative potential. This enhancement is attributed to the increased electroactive surface area provided by the TiO_2_NP and their synergistic effect in facilitating faster electron transfer between the redox probe and the electrode surface.

To further validate the interfacial properties and charge transfer kinetics, EIS measurements were performed, and the results are presented as Nyquist plots in Figure 7b. The plots consist of a semicircular portion at higher frequencies, representing the charge transfer resistance *R*_ct_, followed by a linear portion at lower frequencies, corresponding to the diffusion-limited process (Warburg impedance). The Nyquist plot for the bare SPE displays a significantly larger semicircle diameter, indicating a higher *R*_ct_ (250 Ω). In contrast, the SPE@TiO_2_ shows a much smaller semicircle, with an *R*_ct_ value reduced to approx. 110 Ω.

### Sensitivity studies: calibration and detection limits

**Figure 8.**
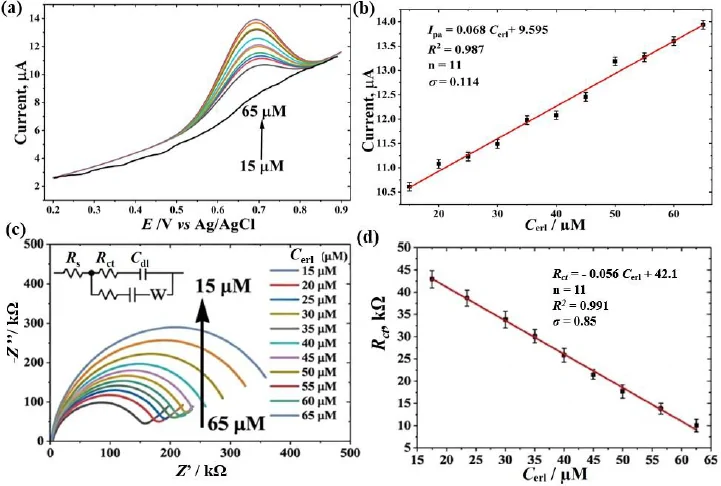
(a) Electrochemical characterization of ERL detection. DPV showing the ERL oxidation peak current increases with concentration (15 to 65 μM) at a scan rate of 50 mV s^-1^; (b) calibration curve showing a linear response of peak current *vs.* ERL concentration described by *I*_pa_ = 0.068 *C*_erl_ + 9.595 (*R^2^* = 0.987, *n* = 11, *σ* = 1.14); (c) Nyquist plots for different ERL concentrations, showing the decrease in *R*_ct_ as the concentration increases and (d) plot of *R_ct_* vs. concentration of ERL showing a linear decrease described by *R_ct_* = -0.56 *C*_erl_ + 42.1 (*R^2^* = 0.991, *n* = 11, *σ* = 0.85)

DPV responses of TiO_2_NP@SPE were recorded for ERL concentrations from 15 to 65 μM in 0.1 M PB (pH 7.4) (Figure 8a). A well-defined anodic peak near 0.65 V *vs.* Ag/AgCl was observed, and the peak current increased linearly with ERL concentration across the entire range studied. The resulting calibration plot (Figure 8b) yielded the linear equation *I*_pa_ = 0.068 *C*_erl_ + 9.595, with a coefficient of determination *R*^2^ = 0.987 (*n* = 11), as in Figure 8b confirming excellent linearity. The residual standard error of the regression was *σ*_reg_ = 1.14 μA (as shown in Figure 8b), which reflects the scatter of experimental data points about the fitted line. LOD and LOQ were calculated using the standard blank-based expressions LOD = 3*σ*_blank_/*m* and LOQ = 10*σ*_blank_/*m*, where *m* = 0.068 μA μM^-1^ is the calibration slope and *σ*_blank_ = 0.2075 μA is the standard deviation of the blank current measured independently from replicate blank recordings (*n* = 6) in 0.1 M PB (pH 7.4) in the absence of analyte. This yielded an LOD of 9.15 μM and an LOQ of 30.52 μM. The area-normalised sensitivity, calculated by dividing the raw slope by the electroactive surface area of the TiO_2_NP@SPE (0.0383 cm^2^), was 1.775 μA μM^-1^ cm⁻^2^.

The comparison of the analytical performance reveals that the TiO_2_NP@SPE provides a superior catalytic environment for ERL detection. While the bare SPE exhibited a raw calibration slope of (0.152 μA μM^-1^), the modified electrode demonstrated an area-normalized sensitivity of (1.775 μA μM^-1^ cm⁻^2^), marking a substantial 10.6-fold increase over the bare electrode’s normalized response (0.167 μA μM^-1^ cm⁻^2^). Although the raw slope of the modified sensor (0.068 μA μM^-1^) is numerically lower than that of the bare electrode, the significant gain in area-normalized sensitivity confirms that the TiO_2_NPs provide a high degree of intrinsic electrocatalytic enhancement, thereby fundamentally improving charge-transfer efficiency at the electrode interface.

Despite this significant gain in sensitivity, the LOD for TiO_2_NP@SPE (9.15 μM) is higher than that of the bare SPE (LOD = 0.03 μM), a result that is primarily attributable to an increase in *σ*_blank_ rather than any deficiency in sensitivity. Surface modification with TiO_2_ nanoparticles substantially increases the double-layer capacitance (*C*_dl_) of the electrode, which raises the non-faradaic background current and its associated fluctuations. Even a proportionally larger increase in *σ*_blank_ relative to m will shift the detection limit to a higher concentration. Furthermore, manual fabrication of the nanoparticle-modified electrode may introduce surface inhomogeneities or adsorbed impurities that introduce additional variability into the blank response. Together, these factors explain the superior sensitivity and a higher LOD can coexist in the modified electrode system [63-65].

The EIS linearity plot (Figure 8c) demonstrates a strong inverse linear relationship between charge transfer resistance (*R_ct_*) and erlotinib concentration across the range of 15-65 μM, described by the equation *R*_ct_ = −0.56 *C*_erl_ + 42.1, with an excellent coefficient of determination (*R*^2^ = 0.991) as in Figure 8d, indicating high reliability of the sensor response. As the concentration of ERL increases, the *R*_ct_ values decrease consistently, suggesting that the progressive binding of ERL molecules to the electrode surface facilitates charge transfer kinetics, likely by disrupting the blocking layer or altering the interfacial properties of the modified electrode. The low standard deviation (*σ* = 0.85) across 11 data points confirms the reproducibility and precision of the measurements, validating the biosensor's analytical performance for quantitative detection of ERL within the tested concentration range.

### Selectivity studies

The selectivity of the TiO_2_NP@SPE was rigorously evaluated against common physiological ions, sugars, neurotransmitters, and metabolic byproducts. As summarized in Table 1, the sensor demonstrated high tolerance toward inorganic ions (Mg^2+^, Na^+^, K^+^ and Ca^2+^) at concentrations as high as 0.1 M, with signal deviations remaining ≤ 0.5 %. Similarly, the addition of common sugars (glucose, sucrose, and maltose at 8.93 mM) resulted in a negligible signal change of -0.21 %, -0.24 % and -0.3 % respectively. Furthermore, key biological interferents, including dopamine (2 μM), urea (1.31 mM) and uric acid (0.5 mM), yielded minor current fluctuations of +1.0, +0.98 and +1.1 %, respectively. These results indicate that the TiO_2_NP@SPE platform maintains high analytical integrity in the presence of high-concentration physiological matrices.

**Table 1.** Influence of potential interferents on the voltammetric response of 22 μM ERL at TiO_2_NP@SPE.

Interferent	Concentration, mM	Signal change, %
Mg^2^⁺	100	+0.01
Na⁺	100	+0.5
K⁺	100	+0.01
Ca^2^⁺	100	+0.01
Glucose	8.93	−0.21
Sucrose	8.93	−0.24
Maltose	8.93	−0.30
Dopamine	0.002	+1.0
Urea	1.31	+0.98
Uric acid	0.5	+1.1

**Figure 9.**
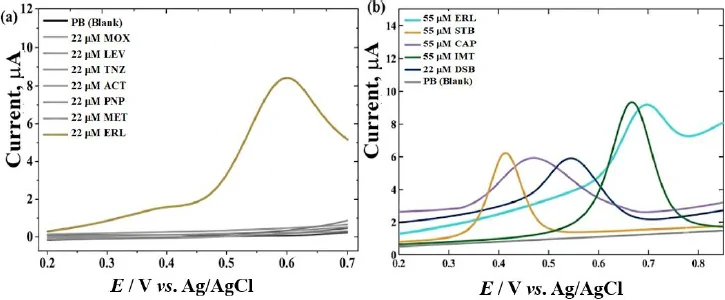
Selectivity and interference studies of the electrochemical sensor: (a) DPVs of the sensor in 0.1 M PB containing 22 μM ERL compared to 22 μM concentrations of various pharmaceutical interferents (MOX, LEV, TNZ, ACT, PNP and MET), showing a distinct faradaic response only for ERL and (b) DPV responses for 55 μM of competing anti-cancer drugs (ERL, STB, CAP, IMT) and 22 μM DSB, demonstrating the resolution of oxidation peaks for multi-drug analysis

The selectivity of the TiO_2_NP@SPE sensor was systematically evaluated against a diverse panel of co-administered pharmaceuticals and structurally related anticancer agents. As depicted in Figure 9a, common co-administered drugs including fluoroquinolone antibiotics MOX, ACT, TNZ, LEV, PNP and MET, each at 22 μM, exhibited no discernible faradaic response across the entire potential window of 0.2-0.7 V, remaining virtually indistinguishable from the blank phosphate buffer baseline. In sharp contrast, 22 μM ERL produced a well-defined, prominent oxidation peak at approximately 0.62 V with a peak current exceeding 11 μA, unambiguously demonstrating the inherent electrochemical inactivity of these common interferents at the TiO_2_NP@SPE surface. The relative standard deviation (RSD) of 1.9 % obtained in the presence of these non-electroactive interferents further confirms that the sensor response to ERL remains essentially unaffected by their presence.

The selectivity of the TiO_2_NP@SPE sensor was evaluated against structurally related anticancer agents that are frequently co-administered with erlotinib in clinical settings. As shown in Figure 9b, at a concentration of 55 μM, anticancer drugs including sunitinib (STB), capecitabine (CAP), and imatinib (IMT) displayed distinct oxidation peaks at approximately 0.42, 0.48 and 0.65 V, respectively, all of which are well-resolved from the characteristic ERL oxidation peak observed at ~0.68 V. Similarly, dasatinib (DSB) at 22 μM exhibited a broad redox response cantered around 0.55-0.60 V, which remains distinguishable from the ERL signal. PB confirmed negligible background current throughout the potential window. Notably, although these electroactive anticancer interferents produce their own faradaic responses, their peak potentials are sufficiently separated from that of ERL to prevent any meaningful signal overlap. RSD of 2.2 % obtained in the presence of these anticancer interferents remains well within the acceptable analytical threshold of 5 %, confirming that despite minor ionic strength variations or surface adsorption dynamics induced by co-existing electroactive species, the TiO_2_NP@SPE platform maintains robust electro-selectivity for the accurate and reliable detection of erlotinib in complex therapeutic matrices.

### Reproducibility and stability

**Figure 10.**
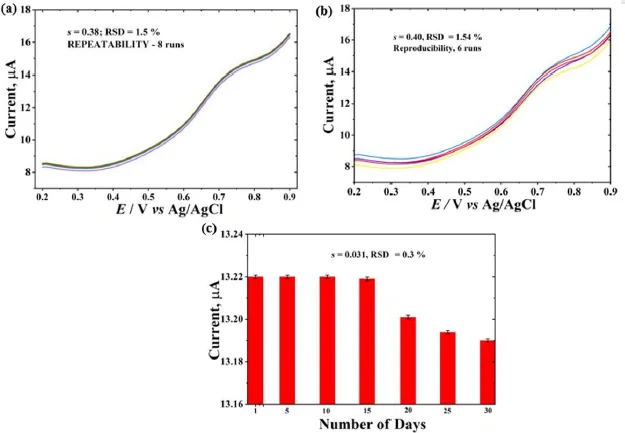
Evaluation of sensor performance for 55 μM ERL detection. (a) Intra-electrode repeatability (*n* = 8), (b) inter-electrode reproducibility (*n* = 6) using TiO_2_NP@SPE in 0.1 M PB (pH 7.4) and (c) Storage stability profile showing the peak current retention over a 30-day period. Error bars represent the standard deviation of triplicate measurements

The repeatability of the TiO_2_NP@SPE was assessed by comparing DPV responses of 8 runs on a single TiO_2_NP@SPE with 55 μM ERL (Figure 10a).

RSD was approximately 1.5 %, indicating excellent repeatability. The reproducibility of the TiO_2_NP@SPE was assessed by comparing DPV responses of 6 independently fabricated TiO_2_NP@SPEs with 55 μM ERL (Figure 10b). RSD was approximately 1 %, indicating excellent batch-to-batch reproducibility.

The long-term stability of the TiO_2_NP@SPE was investigated over 30 days under ambient storage conditions (Figure 10c). The sensor retained approximately 99.9 % of its initial current response after 15 days and 97.7 % after 30 days, with the gradual decline beyond day 15 attributed to slow degradation of the TiO_2_NP film upon prolonged ambient exposure, as reflected by the overall RSD of 0.3 % (*s* = 0.031) across the entire 30-day monitoring period. It should be noted that the influence of environmental variables such as temperature fluctuations and humidity on sensor stability was not investigated in the present study and remain outside the scope of this work; these aspects warrant systematic evaluation in future studies to fully establish the storage robustness of the fabricated sensor under varied real-world conditions.

### Recovery studies in human serum

The practical applicability of the TiO_2_NP@SPE was evaluated by conducting recovery studies in two human serum samples spiked with ERL at 33, 44, and 55 μM. Both intra-day and inter-day analyses were performed (n=6 for each), and the results are presented in Table 2.

**Table 2.** Recovery results of ERL in human serum samples using TiO_2_NP@SPE

Added *C* / μM	Measured *C* / μM ± SD	Recovery, %	RSD, %	*t* value	Measured *C* / μM ± SD	Recovery, %	RSD, %	*t* value
	Intra-day analysis - sample 1				Inter-day analysis - sample 1			
33	33.48 ± 0.72	101.45	2.15	0.48	32.81 ± 0.86	99.42	2.62	0.66
44	43.62 ± 0.91	99.14	2.09	0.52	44.51 ± 1.07	101.56	2.40	0.71
55	54.21 ± 1.08	98.56	1.99	0.57	55.67 ± 1.32	101.22	2.37	0.78
	Intra-day analysis - Sample 2				Inter-day analysis - Sample 2			
33	32.69 ± 0.68	99.06	2.08	0.46	33.74 ± 0.89	102.54	2.64	0.69
44	44.18 ± 0.95	100.41	2.15	0.54	43.36 ± 1.02	98.55	2.35	0.73
55	55.42 ± 1.11	100.76	2.02	0.59	54.38 ± 1.29	98.87	2.37	0.81

*n* = 6, 95 % confidence, tabulated *t* = 4.032

For serum sample 1, intra-day recoveries ranged from 98.56 to 101.45 % (RSD: 1.99 to 2.15 %), and inter-day recoveries were 99.42-101.56 % (RSD: 2.37 to 2.62 %). For serum sample 2, intra-day recoveries were 99.06 to 100.76 % (RSD: 2.02 to 2.15 %) and inter-day recoveries were 98.55 to 102.54 % (RSD: 2.35 to 2.64 %). These results demonstrate that the TiO_2_NP@SPE accurately determines ERL in complex biological matrices with no significant matrix interference. Student’s *t*-test was applied (*n* = 6, *df* = 5, 95 % confidence). The calculated *t*-values were lower than the tabulated *t*-value (4.032), indicating no statistically significant difference between the measured and nominal concentrations.

### Comparison with literature

Table 3 summarizes a comprehensive comparison of the TiO_2_NP@SPE sensor with previously reported methods for ERL detection, encompassing both conventional chromatographic techniques and electrochemical approaches. Among the chromatographic methods, LC-MS/MS [66], UPLC-MS/MS [67] and HPLC-MS/MS [69,69] have been widely employed for ERL quantification in human plasma, offering broad linear ranges (10 to 5000 ng L^-1^) with low detection limits. Capillary electrophoresis [70] has similarly been applied to urine matrices, demonstrating linearity over the range of 0.15 to 20 mg L^-1^. While these techniques provide excellent sensitivity and selectivity, they are inherently limited by their requirement for expensive instrumentation, skilled operators, extensive sample pre-treatment, and prolonged analysis times, rendering them unsuitable for rapid point-of-care or routine clinical monitoring.

**Table 3.** Comparison of TiO_2_NP@SPE with reported methods for ERL detection

Method	Material	Matrix	Linear range, μM	LOD, nM	Ref.
DPV	Bare SPE	Human serum, urine, tablet	2-34	30	[39]
AdSSWV	β-CD NPs/GCE	Serum, urine	0.001 to 8.000	1.07	[60]
LC-MS/MS	-	Human plasma	0.025 to 12.710	—	[66]
UPLC-MS/MS	-	Human plasma	0.025 to 12.700	—	[67]
LC-MS/MS	-	Human plasma	0.025 to 12.700	—	[68]
HPLC-MS/MS	-	Human plasma	0.254 to 5.090	—	[69]
Capillary electrophoresis	-	Human urine	0.38 to 50.9	—	[70]
DPV	MWCNT/PUFIX/HF-PGE	Nail, urine	7.70to 142.00	110×10^3^	[71]
DPV	N-GQdot/CuNPs/PANI@GO-GCE	Serum, urine	0.001 to 35.000	712×10^3^	[72]
DPV	DNA/AuPt/p-L-Met/SPE	Human serum	0.032×10^-3^ to 10^-3^	0.009	[73]
DPV	MIP-pyrogallol/SPCE	Human serum	0.05×10^-3^ to 800×10^-3^	0.016	[74]
DPV	CuBO_2_ nanosheets/GQDs/GCE	Human serum	1.0 to 60.0	500	[75]
DPV	TiO_2_NP@SPE	Human Serum	15 to 65	9150	This work

UPLC-MS/MS - Ultra Performance Liquid Chromatography-Tandem Mass Spectrometry; HPLC-MS/MS - High-Performance Liquid Chromatography-tandem mass spectrometry; AdSSWV - adsorptive stripping square wave voltammetry; β-CD NPs/GCE - β-cyclodextrin nanoparticles/glassy carbon electrode; MWCNT/PUFIX/HF-PGE - multi-walled carbon nanotube/polyurethane/hollow fiber-pencil graphite electrode; N-GQdot/CuNPs/PANI@GO-GCE - nitrogen-doped graphene quantum dots/copper nanoparticles/polyaniline@graphene oxide-glassy carbon electrode; DNA/AuPt/p-L-Met/SPE - DNA/gold-platinum bimetallic nanoparticles/poly-l-methionine/screen-printed electrode; MIP-pyrogallol/SPCE - molecularly imprinted polymer (pyrogallol-based)/screen-printed carbon electrode; CuBO_2_ nanosheets/GQDs/GCE s- cuprous delafossite copper borate (CuBO_2_) nanosheets/graphene quantum dots/glassy carbon electrodes

Electrochemical methods have emerged as attractive alternatives owing to their simplicity, low cost, and miniaturization potential. Among the reported electrochemical sensors, the β-CD NPs/GCE-based AdSSWV sensor [60] achieved an impressive LOD of 1.07 nM with a linear range of 0.001 to 8.000 μM in serum and urine, benefiting from the host-guest recognition properties of β-cyclodextrin. The MWCNT/PUFIX/HF-PGE platform [71] reported a linear range of 7.70 to 142.00 μM with an LOD of 0.11 μM in nail and urine samples. The DPV-based sensor using N-GQdot/CuNPs/PANI@GO-GCE [72] delivered a similarly low LOD of 0.712 nM over a range of 0.001 to 35.000 μM, attributable to the synergistic electrocatalytic activity of the complex nanohybrid architecture. The DNA/AuPt/p-L-Met/SPE biosensor [73] demonstrated picomolar-level detection in human serum through the integration of bimetallic nanoparticles and a DNA recognition layer. The MIP-pyrogallol/SPCE sensor [74] achieved an ultralow LOD of 0.016 nM (0.000016 μM) across a wide dynamic range of 0.05 to 800.00 nM in serum, exploiting the molecular recognition capability of the imprinted polymer. The CuBO_2_ nanosheets/GQDs/GCE sensor [75] provided a linear range of 1.0 to 60.0 μM with an LOD of 0.5 μM in human serum, while the bare SPE sensor [39] offered a linear range of 2 to 4 μM with an LOD of 0.03 μM across multiple matrices including serum, urine, and tablet formulations.

Although several of the above electrochemical sensors exhibit lower LOD values, they invariably rely on elaborate multi-step synthesis of complex nanohybrids, molecularly imprinted polymers, or bioreceptor functionalization, which significantly increases fabrication complexity, cost, and batch-to-batch variability. In contrast, the TiO_2_NP@SPE sensor proposed in this work achieves an LOD of 9.15 μM within a clinically relevant linear range of 15 to 65 μM, which encompasses the therapeutically significant ERL plasma concentration window, without necessitating intricate surface modification procedures. The screen-printed electrode platform inherently ensures disposability, portability, and low cost, while the TiO_2_ nanoparticle modification is achieved through a straightforward single-step process. Furthermore, the oxidation peak potential of 0.66 V observed for ERL at TiO_2_NP@SPE is notably lower than those reported for GCE-based sensors operating at 0.94 and 1.5 V, reflecting the superior electrocatalytic activity of the modified surface toward ERL oxidation and enabling measurements with reduced interference from co-existing electroactive species. Collectively, these attributes position the TiO_2_NP@SPE as a practically viable, clinically applicable, and user-friendly platform for ERL therapeutic drug monitoring.

## Conclusions

This study demonstrates that modification of a disposable screen-printed electrode with TiO_2_ nanoparticles yields a highly sensitive electrochemical sensor (TiO_2_NP@SPE) for the determination of erlotinib, with SEM-EDAX, FTIR, and XRD characterization confirming successful nanoparticle deposition. The sensor exhibited diffusion-controlled oxidation kinetics with a LOD of 9.15 μM, LOQ of 30.52 μM and sensitivity of 1.775 μA μM^-1^ cm⁻^2^, alongside excellent selectivity against physiological interferents and structurally related anticancer agents, and intra- and inter-day serum recovery of 98.56 to 102.54 % (RSD <2.7 %). Despite the improved sensitivity, the TiO_2_NP modification resulted in a higher LOD compared to the bare SPE, owing to elevated background capacitance inherent to nanomaterial-modified electrode surfaces. Future work will explore TiO_2_-carbon nanotube or molecularly imprinted TiO_2_ composite modifications to synergistically lower the detection limit and enhance selectivity, alongside integration into a miniaturized lab-on-chip platform for on-chip sample preparation and point-of-care therapeutic drug monitoring of erlotinib in clinical settings.
